# Emotions in Deaf and Hard-of-Hearing and Typically Hearing Children

**DOI:** 10.1093/deafed/enab022

**Published:** 2021-07-29

**Authors:** Yung-Ting Tsou, Boya Li, Adva Eichengreen, Johan H M Frijns, Carolien Rieffe

**Affiliations:** Unit of Developmental and Educational Psychology, Institute of Psychology, Leiden University, Leiden, The Netherlands; Unit of Developmental and Educational Psychology, Institute of Psychology, Leiden University, Leiden, The Netherlands; Unit of Developmental and Educational Psychology, Institute of Psychology, Leiden University, Leiden, The Netherlands; Center for Disability Studies, The Paul Baerwald School of Social Work and Social Welfare, the Hebrew University of Jerusalem, Jerusalem, Israel; The E. Richard Feinberg Department of Child and Adolescent Psychiatry, Schneider Children's Medical Center, Petah Tikva, Israel; Department of Otorhinolaryngology and Head & Neck Surgery, Leiden University Medical Center, Leiden, The Netherlands; Leiden Institute for Brain and Cognition (LIBC), Leiden, The Netherlands; Unit of Developmental and Educational Psychology, Institute of Psychology, Leiden University, Leiden, The Netherlands; Department of Human Media Interaction, Faculty of Electrical Engineering, Mathematics and Computer Science, University of Twente, Enschede, The Netherlands; Department of Psychology and Human Development, Institute of Education, University College London, London, UK

## Abstract

For deaf and hard-of-hearing (DHH) children living in an environment where their access to linguistic input and social interactions is compromised, learning emotions could be difficult, which may further affect social functioning. To understand the role of emotion in DHH children’s social life, this study investigated emotional functioning (i.e., emotion recognition, empathy, emotion expression), and its relation with social functioning (i.e., social competence and externalizing behaviors), in 55 DHH children and 74 children with typical hearing (aged 3–10 years; *M*_age_ = 6.04). Parental reports on children’s emotional and social functioning and factors related to DHH children’s hearing were collected. Results showed similar levels of emotional and social functioning in children with and without hearing loss. Use of auditory intervention and speech perception did not correlate with any measures in DHH children. In both groups, higher levels of empathy related to higher social competence and fewer externalizing behaviors; emotion recognition and positive emotion expression were unrelated to either aspect of social functioning. Higher levels of negative emotion expression related to lower social competence in both groups, but to more externalizing behaviors in DHH children only. DHH children in less linguistically accessible environments may not have adequate knowledge for appropriately expressing negative emotions socially.

##  

Social interaction involves a dynamic, reciprocal process of stimulation and response between individuals, during which emotions work as oil for engines that facilitate the process (Frijda & Mesquita, 1998; Levenson, 1999; Payton et al., 2000). How people respond to others’ emotions thus have profound influence on their communication and relationships with others. This link between affective responses and their effects on interpersonal relationships is learned by children gradually through overhearing, observing, and participating in social interactions (Saarni, 1999). Disruptions in learning this link through social interactions could result in disruptions in social functioning (e.g., Deneault & Ricard, 2013; Eisenberg et al., 1995; Klein et al., 2018; Wilson, Berg, Zurawski, & King, 2013), which involves the skills and behaviors with which children initiate and maintain their relationships with other people (Arnold et al., 2012; Eisenberg et al., 2000; Wiefferink, Rieffe, Ketelaar, & Frijns, 2012). Being deaf or hard-of-hearing (DHH) in an environment where most significant others use spoken language during early childhood is likely to cause such disruptions. Problems with social functioning, such as externalizing behaviors and peer problems, are more often observed in DHH children who live in a predominantly hearing environment where their access to linguistic input is compromised, compared with children with typical hearing (TH) (e.g., Chao et al., 2015; Dammeyer, 2010; Fellinger, Holzinger, Sattel, & Laucht, 2008; Hoffman, Cejas, & Quittner, 2016; Van Eldik, Treffers, Veerman, & Verhulst, 2004) and compared with DHH children who have DHH parents (e.g., Meadow, 2005; Polat, 2003) or those who are in frequent contact with DHH peers (e.g., Marschark et al., 2012; Wolters, Knoors, Cillessen, & Verhoeven, 2011). However, little is known regarding the extent to which these DHH children’s social functioning is associated with their emotional responses. In the current study, we aimed to narrow this gap by investigating three aspects of emotional functioning involved in social interactions (i.e., emotion recognition, empathy, and emotion expression) in DHH children living in less linguistically accessible environments and in their peers with TH, and how these aspects are related to their social functioning. Below, we reviewed the studies that focused on DHH children who live in environments where their access to language input is compromised (hereafter as DHH children), including those with hearing parents, using oral language as the primary communication mode, and/or attending mainstream schools.

Recognizing others’ emotions allows people to collect the information required for subsequent decisions on responses to social situations. Failing to correctly recognize others’ emotion could lead to maladaptive responses to the situation (Crick & Dodge, 1994; Lemerise & Arsenio, 2000), which was observed in children with aggressive behavior who tended to interpret others’ emotional expressions as hostile (De Castro, Merk, Koops, Veerman, & Bosch, 2005). In regard to DHH children’s ability to recognize emotions, mixed findings have been reported. In preschool years, DHH children showed difficulties matching and labeling different facial expressions of emotions, as compared with their peers with TH (Most & Michaelis, 2012; Wang, Su, Fang, & Zhou, 2011; Wang, Su, & Yan, 2016; Wiefferink, Rieffe, Ketelaar, De Raeve, & Frijns, 2013), yet parents’ ratings on DHH children’s overall emotion recognition were on par with those on children with TH (Ketelaar, Rieffe, Wiefferink, & Frijns, 2013). In studies that included school-aged children, DHH children either showed comparable performances or experienced more difficulties than their peers with TH in facial emotion recognition tasks (Dyck et al., 2004; Wang, Wang, & Hu, 2019; Ludlow et al., 2010; Ziv, Most, & Cohen, 2013). The discrepancies might be attributed to the context in which children were evaluated: parent reports (Ketelaar et al., 2013) gave a more positive picture than experimental tasks (Most & Michaelis, 2012; Wang et al., 2011, 2016; Wiefferink et al., 2013), while paper-based experimental stimuli (Ziv et al., 2013) produced more positive results than computer-based stimuli (Dyck et al., 2004; Wang et al., 2019; Ludlow et al., 2010). Moreover, DHH children’s ability to recognize others’ basic emotions was found to associate with their social competence, while this relation was not observed in children with TH (aged 1–6 years; Ketelaar et al., 2013). Possibly, preschool DHH children depend on their understanding of basic emotions to decide how they should behave in a social context, while their peers with TH may have developed other skills to assist this process, such as the understanding of more complex emotions and of others’ desires and beliefs (Izard et al., 2001; Wellman et al., 2001). One study further indicated that DHH youths (aged 13–21 years) misinterpreted the emotions in social situations more often than their peers with TH and subsequently responded with more aggressive behaviors or crying (Torres, Saldaña, & Rodríguez-Ortiz, 2016).

Sharing the emotional state of other people—a capacity known as empathy—helps create social bonding. Empathy allows people to feel what others are feeling, understand others’ emotion, and respond to the emotion with proper affects and actions such as comforting and helping (Hoffman, 1990; Rieffe, Ketelaar, & Wiefferink, 2010). As a result, people with higher levels of empathy have more friends and show more acceptance toward peers (Eisenberg, Eggum, & DiGiunta, 2010; Zhou et al., 2002) and do less harm to other people (Mayberry & Espelage, 2007; Pursell, Laursen, Rubin, Booth-LaForce, & Rose-Krasnor, 2008). According to parent reports, similar levels of empathy were found in children with and without hearing loss during preschool years (aged 1–6 years; Ketelaar et al., 2013; Ketelaar, Wiefferink, Frijns, & Rieffe, 2017). Yet in the stages where peer interactions become increasingly important, DHH children (aged 4–14 years) were rated lower on empathy by their teachers than children with TH (Peterson, 2016). No evidence has shown that the association between empathy and social functioning is different between children with and without hearing loss (Ketelaar et al., 2013).

Expressing emotions helps people to send specific information to their interaction partners (Levenson, 1999). It shows the other(s) not only what is important to the individual, but also what the sender wants to achieve within the relationship. When a person approaches a friend with anger, they are signaling to the friend that there is an issue which needs to be solved or it might negatively affect their relationship. Yet, frequent or prolonged expression of negative emotions is harmful to social participation and may even be considered a risk factor in young children for developing externalizing behaviors (Liew, Eisenberg, & Reiser, 2004; Sallquist et al., 2009; Wiefferink et al., 2012). Contrarily, more expressions of positive emotions are in general linked to higher levels of social competence (Hayden, Klein, Durbin, & Olino, 2006; Lengua, 2003; Pesonen et al., 2008; Wiefferink et al., 2012), although excessive expressions of positive emotions may also associate with more externalizing behaviors such as hyperactivity and impulsivity (Rothbart, Ahadi, Hershey, & Fisher, 2001).

While the expression of positive emotions does not differ between children with and without hearing loss (aged 1.5–5 years), DHH children (all with a cochlear implant) were reported to show more frequent and more intense negative emotion expression than children with TH (Wiefferink et al., 2012). Rieffe (2012) further showed that in emotion-provoking situations, the intensity of negative emotion expression in DHH children (aged 9.5–13 years; with hearing parents and attending schools for the deaf) did not decrease as much as in children with TH after using a coping strategy (e.g., problem solving or avoidance). It was also found that DHH children (aged 11–12 years; with hearing parents and attending schools for the deaf) more often expressed their anger without constructively explaining the causes, as compared with their peers with TH, who used anger to communicate to the other person who caused the discomfort (Rieffe & Meerum Terwogt, 2006). Moreover, although the excessive expression of negative emotions is related to more externalizing behaviors in children with and without hearing loss alike, only in children with TH are more positive emotion expressions associated with higher levels of social competence (Wiefferink et al., 2012). These group differences in how emotion expression functions in social interactions reflects that DHH children may express emotions in a manner that is less suitable for achieving the goal of maintaining or strengthening their relationships with others (Rieffe & Meerum Terwogt, 2006; Wiefferink et al., 2012).

Investigations on the relations between emotional and social functioning could be of particular rehabilitative relevance to DHH children. As early as the preschool years, difficulties in social functioning have been reported in this population, including more aggressive behaviors, more peer problems, and lower adaptability, as compared with children with TH (Chao et al., 2015; Dammeyer, 2010; Fellinger et al., 2008; Hoffman et al., 2016; Netten et al., 2015; Van Eldik et al., 2004; see Bigler et al., 2019 and Stevenson, Kreppner, Pimperton, Worsfold, & Kennedy, 2015 for a review). Yet, in some studies that focused on DHH preschoolers with a cochlear implant (aged 1–5 years), comparable levels of social functioning were observed (Ketelaar et al., 2013; Ketelaar, Wiefferink, Frijns, Broekhof, & Rieffe, 2015; Netten et al., 2018). This may suggest the benefit of early awareness and support from parents and professionals (as a result of newborn hearing screening), or that social problems are more noticeable when DHH children enter school age. Notably, the hearing loss by itself may not explain the between-group differences in social functioning (Antia, Jones, Luckner, Kreimeyer, & Reed, 2011; Bat-Chava, Martin, & Kosciw, 2005; Fellinger, Holzinger, Beitel, Laucht, & Goldberg, 2009; Huber & Kipman, 2011; Leigh et al., 2015; Patrick et al., 2018; Stevenson et al., 2011; Theunissen et al., 2015; Van Eldik et al., 2004). Compared with children with TH, DHH children growing up in a predominantly hearing environment and surrounded mainly by people using oral language have less opportunities for social interactions and daily incidental learning (i.e., unstructured, unplanned learning), which disrupts their learning of the communicative functions of emotions and in turn could further hinder social functioning (for a review on the effect of environment and access to language on DHH children’s socioemotional development, see: Calderon & Greenberg, 2011; Rieffe, Netten, Broekhof, & Veiga, 2015). Examining how emotional functioning is related to social functioning allows for the factors underlying DHH children’s psychosocial well-being to be disentangled. However, empirical research in this respect is currently lacking.

## Present Study

To successfully navigate the social world, knowing how to respond to others’ emotions are necessary skills. These skills allow an individual to understand others’ concerns and to appropriately express their own concerns to others, which in turn facilitates the person’s social relationships. Given the difficulties in social participation often experienced by DHH children, understanding how emotions function in young DHH children’s daily social life could help us provide support at the earliest possible stage. While a small number of studies have provided us valuable information in this regard, those studies mostly focused on a single aspect of emotional or social functioning. The current study aimed to build on previous studies on this topic by investigating the relation between three aspects of emotional functioning (i.e., emotion recognition, empathy, and emotion expression) and two aspects of social functioning (i.e., social competence and externalizing behaviors) in children with and without hearing loss. We recruited 3- to 10-year-old children because this is the period when typically developing children gradually understand basic and complex emotions and link these emotions to social contexts (Durand, Gallay, Seigneuric, Robichon, & Baudouin, 2007; Harris, 2008). Where possible, we made our hypotheses based on studies that included a similar age range as the current study.

The first aim of this study was to examine the levels of emotional functioning (i.e., emotion recognition, empathy, and emotion expression) and of social functioning (i.e., social competence and externalizing behaviors) in children with and without hearing loss. Based on findings from previous studies on emotional functioning, we expected lower levels of emotion recognition and empathy in DHH children than in children with TH (Peterson, 2016; Wang et al., 2019). Also, we expected DHH children to show more negative emotion expression than their peers with TH, while no difference was expected for positive emotion expression (Wiefferink et al., 2012). In regard to social functioning, we expected lower levels of social competence and more externalizing behaviors in DHH children than in children with TH.

**Figure 1.**
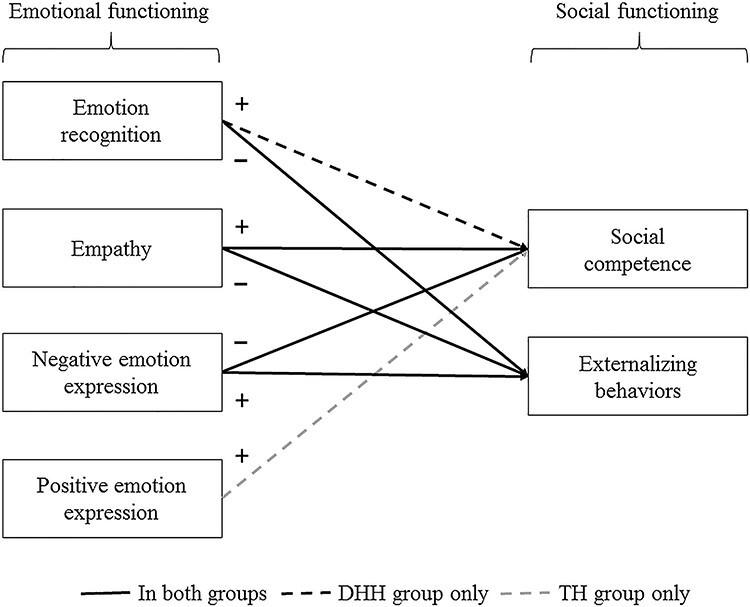
Visual illustration of the hypotheses on the relations between emotional functioning and social functioning. *Note*. Black solid lines represent relations expected to be present in the two groups. Black dotted lines represent relations expected to be present only in deaf or hard-of-hearing (DHH) children. Gray dotted lines represent relations expected to be present only in children with typical hearing (TH).

Second, we aimed to understand how emotions function in social interactions in children with and without hearing loss. Therefore, we investigated to what extent emotional functioning is associated with social functioning, and the moderating role of DHH group membership. We expected that these relations to be in line with previous findings. That is, in both groups, higher levels of empathy and lower levels of negative emotion expression were expected to associate with higher levels of social competence (Ketelaar et al., 2013; Wiefferink et al., 2012). Yet, as Wiefferink et al. (2012) reported, we expected a positive association between positive emotion expression and social competence only in children with TH, but unrelated in DHH children because DHH children living in less linguistically accessible environments might express their positive emotions in a way that is less suitable for maintaining or strengthening social relationships. Also, we expected a positive association between emotion recognition and social competence only in DHH children, but unrelated in children with TH, because DHH children may depend more on their ability to recognize others’ emotions during social interactions (Ketelaar et al., 2013). In regard to externalizing behaviors, we hypothesized lower levels of emotion recognition and empathy, and higher levels of negative emotion expression, to associate with more externalizing behaviors, in the two groups alike (Ketelaar et al., 2013; Wiefferink et al., 2012). We did not expect a relation between positive emotion expression and externalizing behaviors. These hypotheses are visually illustrated in Figure 1.

Additionally, we aimed to understand to what extent each measure of emotional and social functioning is related to hearing-related factors within the DHH group. Previous studies have shown that factors related to degree of hearing loss and habilitation procedures do not associate with social and emotional skills in DHH children who live in an environment where the dominant communication mode is oral and receive early auditory intervention such as a cochlear implant or a hearing aid (Antia et al., 2011; Bat-Chava et al., 2005; Fellinger et al., 2009; Huber & Kipman, 2011; Leigh et al., 2015; Patrick et al., 2018; Stevenson et al., 2011; Theunissen et al., 2015; Van Eldik et al., 2004). Possibly, such factors do not adequately capture these DHH children’s difficulties in access to daily incidental learning and social participation (Leigh et al., 2015; Netten et al., 2018). Therefore, we expected hearing-related factors, including age at receiving auditory intervention (i.e., a cochlear implant or a hearing aid), duration of using the hearing device, type of amplification, and speech perception, to be unrelated to the levels of emotional and social functioning.

## Methods

### Participants

Table 1 shows participant characteristics. A total of 129 children aged 3–10 years from Taiwan were included in the current study. Fifty-five children were DHH (*M*_age_ = 6.24 years, *SD* = 2.11) and wore a cochlear implant (*n* = 50) or a hearing aid (*n* = 5). The other 74 children were with TH (*M*_age_ = 5.90 years, *SD* = 1.80). The children were recruited through a speech pathology and audiology center (the DHH children) and kindergartens/primary schools (the children with TH). None of the children had additional disabilities or diagnoses. Parents were asked to report on additional disabilities or diagnoses. Also, children were excluded when teachers/medical doctors noted cognitive problems or the indices for nonverbal intelligence were two standard deviations below the mean (see below for the indices). All of the children used oral language as the primary communication mode and attended mainstream schools.

**Table 1:** Participant characteristics

	DHH	TH
*N*	55	74
Age, years, mean (*SD*)	6.24 (2.11)	5.90 (1.80)
Gender, female *n* (%)	28 (51%)	44 (59%)
Nonverbal intelligence^a^, mean (*SD*)	9.63 (2.63)	10.46 (2.69)
Parent education level^b^, mean (*SD*)	3.53 (.71)	3.76 (.62)
Net household income^c^, mean (*SD*)	2.44 (1.12)	2.49 (1.03)
Age at auditory intervention, years, mean (*SD*)	2.50 (1.31)	—
Duration of amplification, years, mean (*SD*)	3.74 (1.87)	—
Type of amplification, *n* (%)		
Hearing aid only	5 (9%)	—
Unilateral cochlear implant	35 (64%)	—
Bilateral cochlear implants	15 (27%)	—
Speech perception, percentage correct, mean (*SD*)		
Monosyllabic words	88.90 (11.40)	—
Everyday sentences	86.91 (18.89)	—

*Note*: DHH = deaf and hard of hearing; TH = typical hearing.

^a^For nonverbal intelligence, age-corrected norm scores are presented. The grand population mean is 10 and the standard deviation is 3.

^b^Parental education level: 1 = no/primary education; 2 = lower general secondary education; 3 = higher general secondary education; 4 = college/university.

^c^Net household income: 1 = less than €15,000; 2 = €15,000–€30,000; 3 = €30,000–€45,000; 4 = €45,000–€60,000; 5 = more than €60,000.

The two groups did not differ in terms of age, *t*(127) = −.97, *p* = .333, gender distribution, *χ*^2^(1, *N* = 129) = .94, *p* = .333, nonverbal intelligence, *t*(760) = 1.66, *p* = .098, parental education level, *t*(403) = 1.90, *p* = .059, and net household income, *t*(67) = .34, *p* = .733.^1^ Nonverbal intelligence scores were obtained by averaging the age-corrected standardized scores from Block Design and Matrix subscales of the Wechsler Preschool and Primary Scale of Intelligence Revised Edition (WPPSI-R, for children aged 3–5 years; Wechsler, 1989) or from Block Design and Picture Arrangement subscales of the Wechsler Intelligence Scale for Children Third Edition (WISC-III, for children aged 6–10 year; Wechsler, 1991). Verbal subtests of the WPPSI-R/WISC-III were not conducted because these measures may not accurately reflect the potential of DHH children, who experience incidental learning and receive language exposure differently from the children in the normative sample (Day, Adams Costa, & Raiford, 2015).

This study was part of a larger scale research project in which other variables were included to examine different aspects of social–emotional functioning in children with typical and atypical development. Guardians of the child participants and children older than 7 years signed the informed consent forms before the test procedures. The study protocol and informed consent form were approved by the Psychology Ethics Committee of Leiden University and Chang-Gung Memorial Hospital Ethics Committee for Human Studies.

A total of 153 children were approached for this project. After excluding children whose parents did not return the questionnaires (*n* = 9), who had additional diagnoses or disabilities or nonverbal intelligence two standard deviations below or above the mean (*n* = 14; according to WISC-III/WPPSI-R or indicated by teachers/responsible doctors), and who was accidentally given the questionnaires twice (*n* = 1), a final sample included 129 children. No differences in age, *t*(151) = 1.89, *p* = .058, and nonverbal intelligence, *t*(83) = −1.25, *p* = .216, were observed between the included and excluded samples. Yet, the children excluded from this study had a larger proportion of boys, *χ*^2^(1, *N* = 153) = 4.10, *p* = .043, lower parental educational level, *t*(51) = −3.55, *p* = .001, and lower net household income, *t*(195) = −2.17, *p* = .031, than the children included in the study.^1^ See Supplemental Table 1 for the sample size justification.

### Materials

To address our first and second aims, parental questionnaires for children’s emotional functioning (emotion recognition, empathy, and emotion expression) and social functioning (social competence and externalizing behaviors) were administered at the speech pathology and audiology center or at the child’s school. Parents also provided background information, such as parental education level and household income. To address our third aim, hearing-related data were collected from DHH children’s medical records. In addition, an experimenter administered the nonverbal intelligence tasks (WPPSI-R or WISC-III) to the child in a quiet room. All measures were conducted by the same experimenter, who received training before data collection.

#### Emotional functioning

##### Emotion recognition

The Emotion Acknowledgment subscale (6 items) from the *Emotion Expression Questionnaire* (*EEQ*; 35 items) was used to examine the overall emotion recognition ability. Parents rated how well their children can understand the emotions expressed by them (e.g., “does your child understand when you are angry?”; Rieffe et al., 2010), on a 5-point scale [1 = (almost) never; 5 = (almost) always)]. The internal consistency was good (Table 2). See Supplemental Table 2 for the items in each parent report.

**Table 2:** Psychometric properties and mean scores (standard deviations) of the measures

	*N* items	Scale	Cronbach’s α (*N* sample)	Mean (SD)		*t* value^a^	*p-*value^ab^
				DHH	TH		
Emotion recognition	6	1–5	.84 (127)	3.71 (.66)	3.61 (.74)	−.84	.202
Empathy (all children)	—	0–2	—	1.21 (.29)	1.25 (.34)	.65	.257
Empathy (3–5 years)	19	0–2	.78 (63)	1.07 (.25)	1.07 (.28)		
Empathy (6–10 years)	18	0–2	.85 (60)	1.35 (.27)	1.42 (.29)		
Negative emotion expression	8	1–5	.80 (128)	2.38 (.56)	2.43 (.54)	.50	.308
Positive emotion expression	6	1–5	.73 (128)	3.65 (.63)	3.63 (.54)	−.26	.399
Social competence	8	0–2	.69 (125)	1.47 (.36)	1.52 (.30)	.72	.235
Externalizing problems	9	0–2	.73 (126)	.71 (.38)	.62 (.30)	−1.52	.065

*Note*: DHH = deaf and hard of hearing; TH = typically hearing.

^a^Pooled results after multiple imputations.

^b^One-tailed. Significance level was corrected for multiple testing to *p* < α/6 = .0083.

##### Empathy

We used age-appropriate measures for empathy. For preschool children (aged 3–5 years), the *Empathy Questionnaire* (*EmQue*, 19 items) was used (Rieffe et al., 2010). Parents rated on a 3-point scale (0 = never; 2 = often) in regard to their children’s empathic responses to other people’s emotional displays, such as “when another child is upset, my child needs to be comforted too,” “when another child is angry, my child stops his own play to watch,” and “when another child starts to cry, my child tries to comfort him/her.”

For school-aged children (aged 6–10 years), the *Empathy Questionnaire for Children and Adolescents* (*EmQue-CA*, 18 items) was administered (Overgaauw, Rieffe, Broekhof, Crone, & Güroglu, 2017). The *EmQue-CA* was originally a self-report and was adapted into a parent report for this study by replacing “I” with “my child.” On a 3-point scale (0 = no; 2 = often), parents rated on items such as “when a friend is upset, my child feels upset too,” “my child understands that a friend is ashamed when he/she has done something wrong,” and “if a friend is sad, my child likes to comfort him.”

The internal consistencies were good for both questionnaires (Table 2). *EmQue* and *EmQue-CA* did not differ in their correlations with other measures in this study (see Supplemental Table 3). Also, the distribution of children with and without hearing loss who were rated on *EmQue* was comparable to the distribution of children with and without hearing loss rated on *EmQue-CA*, *χ*^2^(1, *N* = 129) = .01, *p* = .919. Therefore, the total scores from the two questionnaires were combined into one variable for later analyses.

##### Negative and positive emotion expression

We used the Negative Emotion Expression subscale (8 items) and Positive Emotion Expression subscale (6 items) from the *EEQ* (Rieffe et al., 2010). On a 5-point scale, parents scored the frequency, intensity, and duration of their child’s expressions of negative emotions, including anger and sadness, and positive emotions, including happiness and joy, as well as the extent to which the child can calm down from the emotional episode. Example items are “how often does your child show anger?” [1 = (almost) never; 5 = (almost) always] and “is your child easy to calm down when angry?” (1 = very easy; 5 = very difficult). The internal consistencies were good for both subscales (Table 2).

#### Social functioning

The *Strengths and Difficulties Questionnaire* (SDQ; 25 items) is a widely used instrument for measuring children’s social functioning (Goodman, 1997). Parents rated each statement on a 3-point scale (0 = not true; 2 = certainly true). In the current study, we examined two aspects of social functioning: externalizing behaviors and social competence. Externalizing behaviors (9 items) were assessed by the combination of the subscales Hyperactivity (5 items; e.g., “restless, overactive, cannot stay still for long”) and Conduct Problems (4 items; e.g., “often fights with other children or bullies them”). One item from the original Conduct Problems subscale was removed because it conceptually overlapped with the measure for negative emotion expression (“often has temper tantrums or hot tempers”).

To assess social competence (8 items), we combined the subscales Prosocial [4 items; e.g., “shares readily with other children (treats, toys, pencils etc.)”] and Peer Relation (4 items; e.g., “has at least one good friend”; items denoting peer problems were reversely scored). Following Ketelaar et al. (2013), one item from the original Prosocial subscale was removed because it conceptually overlapped with the measures for empathy (“helpful if someone is hurt, upset or feeling ill”). One item from the original Peer Relation was removed because it had a negative correlation with the other items in the merged social competence scale and thus reduced internal consistency (“gets on better with adults than with other children”). The internal consistencies for the two aspects of social functioning were adequate (Table 2).

#### Translation procedures

The Traditional Chinese version of the *SDQ* is readily available (available at http://sdqinfo.org). The Traditional Chinese versions *EEQ*, *EmQue*, and *EmQue-CA* were adapted from the original English questionnaires for the purpose of this study, following a standard back-translation procedure. After the first author translated the English questionnaires to Traditional Chinese, a bilingual translator translated the Traditional Chinese versions back to English. The back-translation was compared with the original version, and language inconsistencies were modified after discussion within the research team.

#### Hearing-related information

Information about DHH children’s hearing history, including age at auditory intervention (i.e., cochlear implant or hearing aid), duration of using the hearing device, and type of amplification, was collected from parents or medical records.

The scores for speech perception assessed during children’s most recent visit to a speech pathology and audiology center were obtained from their medical records. The assessments included a sentence test and a monosyllabic word test. In a quiet room, an audiologist read out a series of sentences or words with her mouth covered, and children were asked to repeat the sentences/words. The sentence tests were developed by Lin et al. (unpublished materials) based on the Central Institute for the Deaf Everyday Sentence test (Silverman & Hirsh, 1955). It includes 15 easy sentences, each with one to seven key words frequently used in daily communication, such as “book” and “car”; and 20 difficult sentences, each with 1–10 key words less frequently used in daily communication, such as “examine” and “dormitory.” The monosyllabic word test includes 25 monosyllabic words that are phonetically balanced (Wang & Su, 1979). The percentage of correctly repeated (key) words was calculated and used in the analyses. Twenty-nine DHH children also received the speech perception in noise tests with a signal-to-noise ratio of +5 dB. These scores are reported in Supplemental Table 4.

**Table 3:** Hierarchical regression analyses for emotional functioning measures on social functioning (pooled results after multiple imputations)

	Social competence (*N* = 129)			Externalizing behaviors (*N* = 129)		
	*b*	*p*	95% CI	*b*	*p*	95% CI
Step 1	*R*^2^ = .25^*^^*^			*R*^2^ = .25^*^^*^		
Age	<.001	.798	[−.003, .002]	.001	.322	[−.001, .004]
Gender	.01	.866	[−.10, .11]	.04	.523	[−.07, .14]
Group	−.04	.404	[−.15, .06]	.09	.105	[−.02, .20]
Emotion recognition	.05	.235	[−.03, .14]	−.01	.793	[−.10, .08]
Empathy	.28	.009	[.07, .48]	−.31	.005	[−.52, −.09]
Negative emotion expression	−.18	<.001	[−.28, −.08]	.22	<.001	[.12, .32]
Positive emotion expression	.01	.815	[−.08, .10]	.01	.771	[−.08, .11]
						
Step 2	∆ *R*^2^ = .04			∆ *R*^2^ = .07^*^		
Age				.002	.165	[−.001, .004]
Gender				.04	.444	[−.06, .15]
Group				−.56	.233	[−1.47, .36]
Emotion recognition				.04	.477	[−.07, .15]
Empathy				−.50	<.001	[−.76, −.25]
Negative emotion expression				.06	.428	[−.08, .19]
Positive emotion expression				.04	.524	[−.09, .17]
Group × Emotion recognition				−.08	.349	[−.26, .09]
Group × Empathy				.33	.099	[−.06, .71]
Group × Negative emotion expression				.32	.002	[.12, .51]
Group × Positive emotion expression				−.06	.542	[−.24, .13]

*Note*: Gender was coded as 0 = male, 1 = female. Group was coded as 0 = typically hearing, 1 = deaf and hard of hearing. 95% CI: 95% confidence interval. Independent variables were considered having an effect when *p* < α/2 = .025. ^*^p < .05; ^*^^*^p < .001 for the change in *R*^2^.

### Statistical Analysis

Statistical analyses were carried out using SPSS 25.0 (IBM Corp., Armonk, NY). To address our first research question, levels of emotional functioning (negative emotion expression, positive emotion expression, emotion recognition, and empathy) and social functioning (social competence and externalizing behaviors) were compared between DHH and TH children using independent *t*-tests. The normality of the variables was checked by inspecting the normal Q-Q plots. While all the variables followed an approximate normal distribution, we observed a violation of the normality assumption in negative emotion expression. Thus, a Mann–Whitney *U* test was also conducted to compare the levels of negative emotion expression between the groups.

Our second research question was tested using hierarchical regression analyses in which the moderating role of group membership in the contribution of emotional functioning to social functioning was examined. In the first step, we entered age, gender (0 = male, 1 = female), group (0 = TH, 1 = DHH), and the four variables for emotional functioning. In the second step, we added the two-way interactions of group with each of the four emotional functioning variables. Continuous independent variables were centered. The assumptions of linear regression (i.e., normality of residuals, homoscedasticity, no multicollinearity, and linearity) were examined and no violations were noted.

When addressing the first two research questions, we conducted the analyses with and without the five children who did not use cochlear implants. Excluding the children without cochlear implants did not change the results (see Supplemental Table 5a and b). Therefore, we included all DHH children in our analyses and report the results accordingly.

Finally, using partial correlation analyses controlling for age, we checked within the DHH group whether the emotional and social functioning variables were related to hearing-related factors. Included factors were age at auditory intervention, duration of using the hearing device, number of cochlear implants (0 = only hearing aids; 1 = unilateral implantation; 2 = bilateral implantation), and word and sentence perception scores.

In the event of multiple testing, Bonferroni correction was applied to adjust the significance level according to the number of analyses. See the notes under each table for the corrected significance level.

#### Missing values and multiple imputations

Due to time constraints, nonverbal intelligence tasks were not administered to five children (4 DHH, 1 TH), and only one of the two nonverbal intelligence tasks was conducted in 24 children (14 DHH, 10 TH). Parental educational levels and net household income were not available for nine (6 DHH, 3 TH) and 22 (16 DHH, 6 TH) children, respectively. In each questionnaire, parents of DHH children (*n* = 3) missed one or two items. Also, speech perception scores were only available for 42 DHH children. Little’s MCAR test showed that the data were missing completely at random (*p*s > .148 for the samples rated on EmQue/EmQue-CA and for the DHH sample). Missing data could lead to biased interpretation and a loss of power given that most statistical methods apply complete case analysis (Donders, Van Der Heijden, Stijnen, & Moons, 2006; Netten et al., 2017) Therefore, we used multiple imputations, a technique that fills in missing data points based on the characteristics of participants and the relations in the dataset with other participants (Azur, Stuart, Frangakis, & Leaf, 2011; Schafer & Graham, 2002; Van Buuren, 2012). The missing values described above were estimated along with age, gender, group membership, and social/emotional functioning scores. Ten imputations were performed (Sterne et al., 2009). Pooled results are reported for all the analyses. The *F* tests for regression model fits (i.e., pooled ∆*R*^2^) on the multiply imputed data were conducted according to the approach and the SPSS macro (*MI-MUL2*) provided by Van Ginkel (2010, 2019).

## Results

### Group Differences

According to parent reports, the children with and without hearing loss had similar levels on all emotional and social functioning measures, *t*s < 1.52, one tailed *p*s > .128 (Table 2). Because negative emotion expression was not normally distributed, we additionally conducted a Mann–Whitney *U* test, which also showed no group difference in the levels of negative emotion expression, *U* = 1874.50, *p* = .443. Post-hoc *t*-tests were conducted to understand if the absence of group differences was related to age. We divided the children into three age groups (see Supplemental Table 6), and no differences between DHH children and children with TH were noted in each age group. Yet note that these post-hoc tests had small sample sizes and thus should be interpreted with caution.

### Relations Between Emotional and Social Functioning

Table 3 shows the results of hierarchical regression models. In the analysis on social competence, lower levels of negative emotion expression, *b* = −.18, *p* < .001, 95% CI [−.28, −.08], and higher levels of empathy, *b* = .28, *p* = .009, 95% CI [.07, .48], contributed to higher levels of social competence. Adding interactions of group with emotional functioning variables did not improve the model fit, ∆*R*^2^ = .04, *F*(4, 115.04) = 1.83, *p* = .129. This suggests that the relations between social competence and each of the emotional functioning measures were not moderated by group. No other effects were observed.

**Figure 2.**
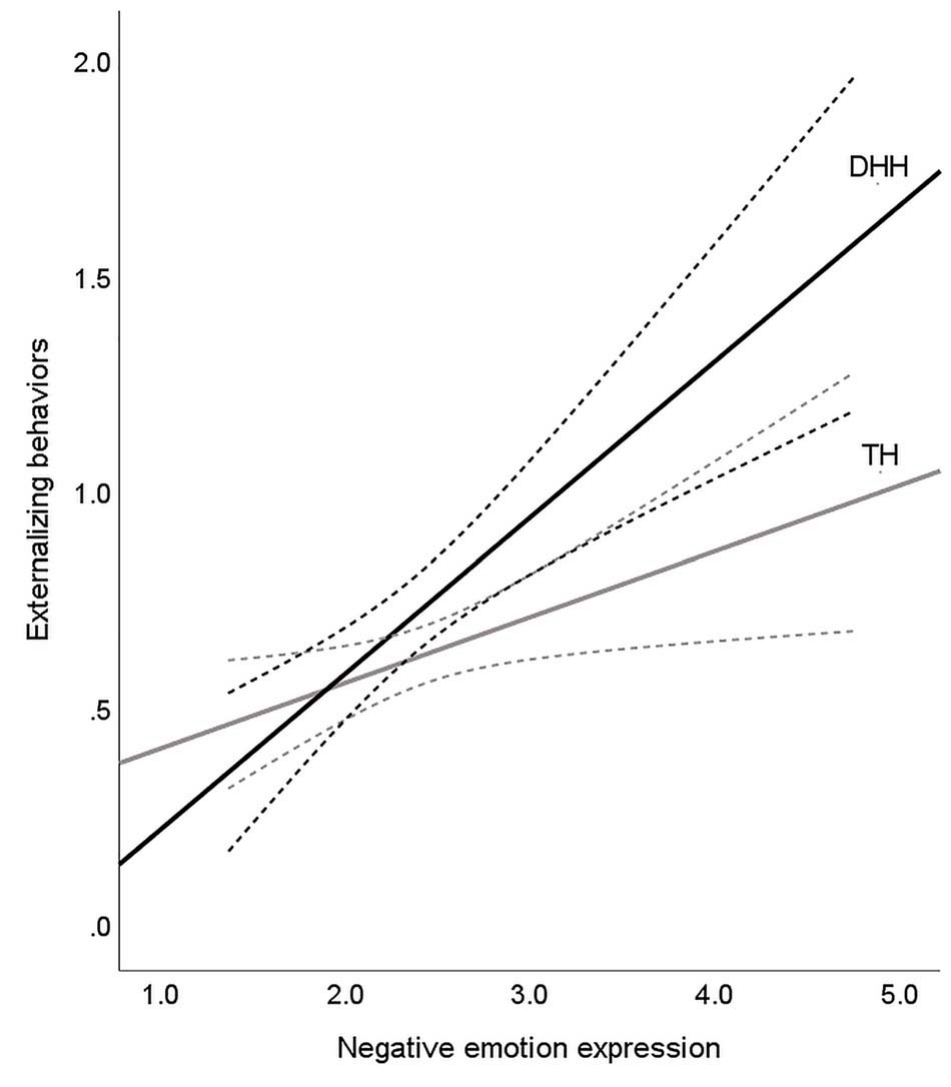
Group status moderates the effect of negative emotion expression on externalizing behaviors. *Note*. Deaf and hard-of-hearing (DHH) children are represented in black; children with typical hearing (TH) are represented in gray. The dotted lines represent 95% confidence interval.

In the analysis on externalizing behaviors, adding group interaction terms in the second step improved the model fit, ∆*R*^2^ = .07, *F*(4, 114.95) = 3.03, *p* = .020. Group membership fully moderated the relations between negative emotion expression and externalizing behaviors: Higher levels of negative emotion expression were associated with more externalizing behaviors only in the DHH group, *b* = .32, *p* = .002, 95% CI [.12, .51], but not in the group with TH, *b* = .06, *p* = .428, 95% CI [−.08, .19] (see Figure 2). Yet, in both groups, lower levels of empathy contributed to more externalizing behaviors, *b* = −.50, *p* < .001, 95% *CI* [−.76, −.25]. No other effects were observed.

### Relations With Hearing-Related Factors

Table 4 shows the correlations between emotional and social functioning variables and hearing-related factors in DHH children, while age was controlled for. None of the correlations reached significance.

**Table 4:** Partial correlations between hearing-related factors and emotional–social functioning measures in DHH children (*n* = 55), controlling for age (pooled results after m ultiple imputations)

	Age at auditory intervention	Duration of amplification	No. cochlear implants	Word perception	Sentence perception
Emotion recognition	.18	−.18	−.28	−.01	.06
Empathy	.04	−.04	.05	.26	.02
Negative emotion expression	−.12	.12	.30	.21	−.19
Positive emotion expression	.04	−.04	.03	−.05	−.07
Social competence	.15	−.15	−.11	.18	.25
Externalizing behaviors	−.24	.24	.09	−.10	−.15

*Note*. Significance level was set at *p* < α/5 = .01.

## Discussion

Emotions serve important interactional functions in daily social life. Recognizing the emotions of others and reacting empathically to them allow people to collect necessary information for evaluating possible response options and to establish closer relationships with others. The expression of emotions allows people to send specific information about one’s attitude, intention, and feelings to interaction partners. The current study examined the relations between emotional and social functioning, in order to deepen our understanding on how emotions function in DHH children’s social life. The levels of emotional and social functioning were similar in DHH children and their peers with TH. In both groups, higher levels of empathy were related to higher levels of social competence and fewer externalizing behaviors. Higher levels of negative emotion expression were associated with lower level of social competence in both groups, but with more externalizing behaviors in DHH children only. Positive emotion expression and emotion recognition were unrelated to social functioning, whether in DHH children or in children with TH. Also, hearing-related factors did not correlate with any of the emotional and social functioning measures. Below, the implications of these findings will be discussed.

Previous studies showed mixed results regarding skills for emotional functioning in DHH children. In our sample, we found DHH children to be on par with their peers with TH. A possible explanation for the absence of group differences is the early detection of hearing loss and the educational system in Taiwan. More than half (*n* = 33; 60%) of the DHH children in this study received newborn hearing screening (launched in 2012) within their first days after birth. Thus, families with these DHH children were aware of the hearing loss and received systematic support very early. This is congruent with some studies that also included DHH children with early detection and a cochlear implant in the Netherlands (Ketelaar et al., 2013, 2017; Netten et al., 2018). Also, mainstream schools provide periodical or regular extra support to DHH children and other children with special needs. These children often start with a tailor-made curriculum that helps them adapt to the pace of mainstream schools (Special Education Transmit Net Taiwan, 2021).

Alternatively, the setting where children were evaluated might have contributed to these positive outcomes. Comparable results between DHH children and children with TH were reported in several other studies that also asked parents to rate the levels of emotional functioning (Ketelaar et al., 2013, 2017; Dirks et al., 2017). However, group differences were often found when DHH children’s emotional functioning was assessed outside the familial setting. For example, a study asked teachers to rate children’s overall empathic responses and found lower levels of empathy in DHH children (Peterson, 2016). Studies that measured emotion recognition in a controlled, experimental setting also noted differences between children with and without hearing loss in their ability to match and label facial emotions (Wang et al., 2011, 2016, 2019; Wiefferink et al., 2013). The discrepant findings suggest that DHH children may still face emotional functioning challenges outside the family circle or that parents overrate their DHH child’s functioning.

Notably, when we linked emotional functioning to the social context, a group difference emerged. Excessive expressions of negative emotions were associated with more externalizing behaviors in DHH children only. This shows that DHH children’s social functioning might be particularly dependent on their expression of negative emotions. Past studies reported that DHH children expressed their negative emotions less strategically than children with TH (e.g., without constructively explaining the cause to interaction partners; Rieffe & Meerum Terwogt, 2006). They also had more difficulties calming themselves down by diverting their attention away from the negative stimuli or by using other coping strategies (Rieffe, 2012; Wiefferink et al., 2012). The more effortful process for controlling negative emotions is likely a result of their lack of familiarity with display rules or coping options toward different social situations. Such kinds of knowledge are primarily learned during social interactions, to which DHH children have limited access (Morris, Silk, Steinberg, Myers, & Robinson, 2007; Saarni, 1999; Thompson, 1994). Moreover, parental overprotection and linguistic over-simplification are often observed between DHH children and their parents with TH (Calderon & Greenberg, 2011; Pinquart, 2013; Vaccari & Marschark, 1997). These leave DHH children with fewer opportunities for learning skills by trial and error, and fewer explanations from parents for more abstract concepts, such as mental states (Dirks, Stevens, Kok, Frijns, & Rieffe, 2020; Moeller & Schick, 2006; Morgan et al., 2014). In turn, DHH children may learn less how to express emotions constructively. Thus, although on average DHH children expressed negative emotions as much as their peers with TH, they may find negative emotions harder to moderate or to explain, when the emotional arousal they experience is high, or when they cannot fully understand a social situation or be understood in the situation (e.g., in a situation novel to them or without a shared language with interaction partners), which further affects their social behaviors. This outcome also implies that when DHH children can effectively display or regulate their negative emotions, the externalizing problems in these children could decrease to a more notable extent than in children with TH. Note that in this study, none of the skills for emotional and social functioning correlated with hearing-related factors. In other words, this potential difficulty for negative emotions might not simply “disappear” when DHH children have more listening experience or perceive speech better. They may need specific considerations from parents and professionals/teachers for gaining more social interactions and mental-state talks.

The cultural background could be another factor that played a role. In this study, children from Taiwan were recruited. Previous cross-cultural studies showed that East Asian youths experienced higher levels of personal emotional arousal than Western counterparts when seeing other people’s emotional displays (Cassels, Chan, Chung, & Birch, 2010; Trommsdorff, 1995). As a result, East Asian DHH children may have to invest an even larger amount of effort to moderate their emotion expressions in a socially favorable manner. Also, cross-cultural studies found that intense expressions of positive emotions were valued more in Western, individualistic cultures than in East Asian, collectivistic cultures (Eisenberg, Liew, & Pidada, 2001; Heine, Lehman, Markus, & Kitayama, 1999; Kitayama, Markus, & Kurokawa, 2000; Tsai, Chentsova-Dutton, Freire-Bebeau, & Przymus, 2002; Tsai, Levenson, & McCoy, 2006). Such a tendency was reflected in studies on Western individuals with TH that showed a positive relation between positive emotion expression and social competence (e.g., Hayden et al., 2006; Lengua, 2003; Pesonen et al., 2008; Wiefferink et al., 2012). However, in this study, positive emotion expressions did not contribute to social competence in children with and without hearing loss, possibly because East Asians tend to balance positive emotion expressions in order to “fit in” to a collectivistic culture (Heine et al., 1999; Tsai et al., 2006).

Importantly, we found that higher levels of empathy were associated with higher levels of social competence and fewer externalizing behaviors, in both groups. Children, who are able to feel what others are feeling and understand what causes others’ emotions, may also be more able to establish bonding with their peers and to know whether their behaviors would cause harm to others (Eisenberg et al., 2010; Mayberry & Espelage, 2007; Pursell et al., 2008; Zhou et al., 2002). This finding thus could be relevant to interventions: Programs that improve children’s empathic responses may help both DHH children and children with TH show more adaptive social behaviors.

### Limitations and Future Directions

This study examined the relations between emotional and social functioning in DHH children. By investigating three aspects of emotional functioning involved in responding to others’ emotions, we gained a clearer understanding of the challenges DHH children have in daily social interactions. Nevertheless, some limitations must be considered when interpreting our results.

First, this study was cross-sectional. Therefore, no causal relations could be drawn from our results. We also could not conclude whether the outcomes would be stable across time, although we did not observe an effect of age in the current study. Future investigations are needed to examine the developmental trajectories and longitudinal associations between emotional and social functioning in DHH children. It should be noted that problems with emotional and social functioning might become more pressing for DHH children in adolescence, when they spend more time with peers, engage in peer activities that require higher levels of verbal sharing and social attunement (Hartup, 1993; Rose & Asher, 1999), and are in more difficult acoustic conditions (e.g., larger classes and more group conversations; Punch & Hyde, 2011; Rieffe et al., 2018).

Second, the emotional functioning measures in this study examined children’s responses to basic emotions. Yet, real-life social situations often involve complex emotions or mixed emotions. Previous studies have shown that DHH adolescents reported shame and guilt less often in social situations that elicited moral emotions (Broekhof, Bos, Camodeca, & Rieffe, 2018), and reported fewer emotions in stories that were designed to trigger more than one emotion (Rieffe, 2012), compared with their peers with TH. For this reason, we still need further studies to understand how DHH children respond to more complex emotions and how such an ability is related to social functioning.

Third, only parent reports were used in this study. To increase the possibility that parents answered accurately and thus to minimize common method biases, we provided clear instructions before each questionnaire and ensured unambiguity in sentence content and structure by asking a team of native researchers to check the translated questionnaires (see Podsakoff, Mac Kenzie, & Podsakoff, 2012, for suggestions on procedural remedies for common method biases). Also, Evans (1985) and Siemsen, Roth, and Oliveira (2009) demonstrated that common method biases do not account for the effects observed in studies designed to test interaction effects. The moderating effect of hearing status found in the current study thus was not at risk to be biased. Nevertheless, future studies are suggested to include multiple methods, such as observations and *in vivo* experiments, to increase ecological validity.

Fourth, the background characteristics of the DHH children should be considered. In this study, we focused on DHH children living in a predominantly hearing environment, surrounded by family members and peers with TH. All these DHH children used oral language as their primary communication mode, and the majority of them had profound hearing loss and a cochlear implant. Therefore, the conclusions of the current study may not extend to DHH children who live in a more linguistically accessible environment, for example, with parents who are skilled in sign language (e.g., see Hoffmeister, 2008; Knoors & Marschark, 2012; Vaccari & Marschark, 1997). Moreover, it should be noted that we considered only children’s nonverbal intelligence, but not their verbal intelligence. Although we checked hearing-related factors and no effects were observed, future studies are suggested to also include speech perception in noisy environment and additional measures for DHH children’s language and communication skills, to examine the extent to which these factors affect the relationship between emotional and social functioning. The heterogeneity in the DHH population is also worth further investigations. For example, a study reported that DHH adolescents in special education were unique in how they regulated emotions (Rieffe et al., 2018). Their use of approach strategies (e.g., problem-solving; seeking social support) was related to negative friendship features, possibly because they approached in a blunt manner, while in mainstreamed DHH adolescents and in their peers with TH, the use of approach strategies was related to positive friendship features. Moreover, the DHH children in our sample did not have additional diagnoses or disabilities. It should be noted that a considerable number of DHH children in the general population do have additional disabilities, which is not only known to be linked to more difficulties with emotional and social functioning (Dammeyer et al., 2010) but also to lower speech and language scores (Van der Straaten et al., 2019). It would be worthwhile to conduct a similar study in a sample of DHH children with additional disabilities. Thus, larger sample sizes will allow to examine individual differences in factors related to hearing, language, and/or educational background as these factors might affect how emotions are experienced and expressed.

Finally, future studies are suggested to assess if the cultural values affect DHH children’s emotional and social functioning. This study was the first to examine the relations between emotional and social functioning in East Asian DHH children. It increases the external validity of the current knowledge about DHH children’s psychosocial development that has been largely built on Western samples. Notably, our results regarding the expression of emotions and its link to social functioning were not consistent with previous research on Western samples. Past studies have shown that individualistic-oriented cultures promote personal uniqueness, while collectivistic-oriented cultures value group harmony (Markus & Kitayama, 1991; Singelis, 1994), and the cultural values influence how emotions are expressed and valued (e.g., Trommsdorff, 1995; Tsai et al., 2006). Although out of the scope of the current study, a cross-cultural design is needed for further research to understand the extent to which these cultural values affect the inclusion of DHH children in their social environment and their socialization experiences.

## Conclusions

This study examined emotional and social functioning in DHH children living in a predominantly hearing environment where their access to linguistic input is often compromised. Differences between DHH children and children with TH were hardly observed in the current study. DHH children recognized the basic emotions displayed by other people, empathically responded to those emotions, and expressed positive and negative emotions as much as their peers with TH. Also, higher levels of empathy were associated with higher levels of social competence and fewer externalizing behaviors in both groups. Only one group difference was found: More negative emotion expressions were related to more externalizing behaviors in DHH children only. DHH children may express their negative emotions less strategically or regulate those emotions less efficiently, possibly because they were unfamiliar with the display rules or coping options toward different social situations. They may also experience more difficulties explaining their negative emotions constructively, for example, addressing the causes of the emotion or the goal of an interpersonal relationship, due to more limited access to linguistic discourses about emotions. It is noteworthy that none of the emotional and social functioning measures in this study were correlated with hearing-related factors. Despite the long-term use of hearing devices and a fairly good ability to perceive speech, producing emotional responses in social interactions could still be an effortful task for some DHH children. Our findings call for closer investigations into the functioning of emotions in DHH children’s daily social life and underscore the need to provide more social interaction opportunities to DHH children for learning the knowledge required for efficient regulation and effective expression of emotions. Moreover, intervention programs that facilitate mental-state talks between DHH children and their meaningful others, especially on the experience of negative emotions, could be beneficial.

## Supplementary Material

DHH_TW_emotion_function_R_Supplements_final_enab022

## Footnotes

The *t*-statistics presented here was based on the multiply imputed dataset, due to missing data in some variables. See the Statistical Analysis section for more details.
