# Supplementary material for: Emotions in Deaf and Hard-of-Hearing and Typically Hearing Children
Source: J Deaf Stud Deaf Educ. 2021 Jul 29;26(4):469–82. doi: 10.1093/deafed/enab022 (PMC8448426; doi:10.1093/deafed/enab022)
Supplement: DHH_TW_emotion_function_R_Supplements_final_enab022 [file dhh_tw_emotion_function_r_supplements_final_enab022.docx]

**Supplemental Table 1**

*Sample size justification*

| Aspect | Explanation |
| --- | --- |
| Time point of power analysis | The sample size for the research project that embeds this study was estimated a priori with a power analysis. |
| Group differences | Previous studies indicated that a difference in emotional functioning between DHH children and children with TH could be observed with small-to-medium effect sizes (Torres et al., 2016; Wiefferink et al., 2013). Thus, a minimum sample size of 82 was required to detect a group difference (d = .4; α = .05; power = .90). |
| Multiple linear regression | Green (1991) provided a rule of thumb of N > 104 + number of independent variable (IV) for estimating sample size required for multiple linear regression. Harris (1985) suggested to have a minimum of 10 participants per IV for regression using six or more IVs. |
| Sample size estimation | A sample size of 115 children was considered necessary (with 4 predictor variables, 4 interactions, and 2-3 control variables). We approached 153 children in total, and 129 were eventually included in this study. |

*Note*. DHH = deaf or hard-of-hearing. TH = typical hearing.

**Supplemental Table 2**

*Items in each parent-report measure*

| Emotion recognition |
| --- |
| 1. Can your child fully acknowledge others' emotions? 2. Does your child see when you are angry? 3. Does your child see when you are happy? 4. Does your child see when you are afraid? 5. Does your child see when you are sad? 6. Does your child see when you are having fun? |
| Empathy (3-5 years old) |
| 1. When another child cries, my child gets upset too. 2. When I make clear that I want some peace and quiet, my child tries not to bother me. 3. When my child sees other children laughing, he/she starts laughing too. 4. My child also needs to be comforted when another child is in pain. 5. When another child starts to cry, my child tries to comfort him/her. 6. When an adult gets angry with another child, my child watches attentively. 7. When another child makes a bad fall, shortly after my child pretends to fall too. 8. When another child gets upset, my child tries to cheer him/her up. 9. My child looks up when another child laughs. 10. When another child is upset, my child needs to be comforted too. 11. When I make clear that I want to do something by myself (e.g. read), my child leaves me alone for a while. 12. When adults laugh, my child tries to get near them. 13. When another child gets frightened, my child freezes or starts to cry. 14. When two children are quarrelling, my child tries to stop them. 15. My child looks up when another child cries. 16. When other children argue, my child gets upset. 17. When another child gets frightened, my child tries to help him/her. 18. When another child is angry, my child stops his own play to watch. 19. When another child cries, my child looks away. 20. When other children quarrel, my child wants to see what is going on. |
| Empathy (6-10 years old) |
| 1. If I am happy, my child also feels happy. 2. My child understands that a friend is ashamed when he/she has done something wrong. 3. If a friend is sad, my child likes to comfort him. 4. My child feels awful when two people quarrel. 5. When a friend is angry, my child tends to know why. 6. My child would like to help when a friend gets angry. 7. If a friend is sad, my child also feels sad. 8. My child understands that a friend is proud when he/she has done something good. 9. If a friend has an argument, my child tries to help. 10. If a friend is laughing, my child also laughs. 11. If a friend is sad, my child understands mostly why. 12. My child wants everyone to feel good. 13. When a friend cries, my child cries himself/herself. 14. If a friend cries, my child often understands what has happened. 15. If a friend is sad, my child wants to do something to make it better. 16. If someone in the family is sad, my child feels really bad. 17. My child enjoys giving a friend a gift. 18. When a friend is upset, my child feels upset too. |
| Negative emotion expression |
| 1. How often does your child show anger? 2. How intense is this usually? 3. How long does it usually last? 4. Is your child easy to calm down when angry? 5. How often does your child show sadness? 6. How intense is this usually? 7. How long does it usually last? 8. Is your child easy to calm down when he is sad? |
| Positive emotion expression |
| 1. How often does your child show happiness? 2. How intense is this usually? 3. How long does it usually last? 4. How often does your child show joy? 5. How intense is this usually? 6. How long does it usually last? |
| Social competence |
| 1. Rather solitary, tends to play alone (R) 2. Has at least one good friend 3. Generally liked by other children 4. Picked on or bullied by other children (R) 5. Gets on better with adults than with other children (R)# 6. Considerate of other people's feelings 7. Shares readily with other children (treats, toys, pencils etc.) 8. Helpful if someone is hurt, upset or feeling ill# 9. Kind to younger children 10. Often volunteers to help others (parents, teachers, other children) |
| Externalizing behaviors |
| 1. Restless, overactive, cannot stay still for long 2. Constantly fidgeting or squirming 3. Easily distracted, concentration wanders 4. Thinks things out before acting (R) 5. Sees tasks through to the end, good attention span (R) 6. Often has temper tantrums or hot tempers# 7. Generally obedient, usually does what adults request (R) 8. Often fights with other children or bullies them 9. Often lies or cheats 10. Steals from home, school or elsewhere |

*Note*: R = reversely scored; # = removed from the analyses, given the reason provided in the main text.

**Supplemental Table 3**

*Correlations of Empathy Questionnaire (EmQue) and Empathy Questionnaire for Children and Adolescents (EmQue-CA) with other study variables (pooled results after multiple imputations)*

|  | Negative emotion expression | Positive emotion expression | Emotion recognition | Social competence | Externalizing behaviors |
| --- | --- | --- | --- | --- | --- |
| EmQue | -.199 | .182 | .475*** | .392** | -.236 |
| EmQue-CA | -.028 | .065 | .589*** | .259*** | -.351** |
| *Fisher’s r to z* | -.963 | .660 | -.885 | .611 | .699 |
| *p* (*z*)^a^ | .336 | .509 | .376 | .541 | .485 |

**p* < .025; ***p* < .01; ****p* < .001 for correlations between EmQue/EmQue-CA and study variables.

^a^ Significance of the z-score after Fisher’s r-to-z transformation, which was applied to compare the strength of correlations.

**Supplemental Table 4**

*Speech perception in noise (signal-to-noise ratio of +5 dB) in 29 DHH children: mean scores, standard deviations, and correlations with study variables.*

|  | Monosyllabic word perception in noise | Sentence perception in noise |
| --- | --- | --- |
| *Descriptive* |  |  |
| Mean (SD), % | 75.45 (13.72) | 84.93 (15.61) |
| Range, % | 44-96 | 39.5-100 |
|  |  |  |
| *Pearson’s correlation* |  |  |
| Emotion recognition, *r* (*p*) | .17 (.367) | .34 (.071) |
| Empathy, *r* (*p*) | .46 (.013)* | .57 (.001)* |
| Negative emotion expression, *r* (*p*) | .05 (.804) | -.15 (.434) |
| Positive emotion expression, *r* (*p*) | .13 (.501) | -.10 (.606) |
| Social competence, *r* (*p*) | .33 (.085) | .08 (.678) |
| Externalizing behaviors, *r* (*p*) | -.16 (.420) | -.05 (.790) |
|  |  |  |
| *Partial correlation controlling for age* |  |  |
| Emotion recognition, *r* (*p*) | .08 (.677) | .27 (.160) |
| Empathy, *r* (*p*) | .26 (.178) | .43 (.022)* |
| Negative emotion expression, *r* (*p*) | .13 (.504) | -.09 (.638) |
| Positive emotion expression, *r* (*p*) | -.01 (.948) | -.28 (.148) |
| Social competence, *r* (*p*) | .38 (.045) | .11 (.582) |
| Externalizing behaviors, *r* (*p*) | -.23 (.242) | -.11 (.571) |

*Note*. Significance level was corrected for multiple testing to * *p* < α/2 = .025.

**Supplemental Table 5a**

*Psychometric properties and mean scores (standard deviations) of the questionnaires after excluding the five children without a cochlear implant*

|  | Cronbach’s α  (*n* sample) | Mean (SD) | | *t* value^a^ | *p* value^ab^ |
| --- | --- | --- | --- | --- | --- |
|  |  | DHH | TH |  |  |
| Emotion recognition | .83 (122) | 3.64 (.62) | 3.61 (.74) | -.29 | .387 |
| Empathy (all children) | -- | 1.22 (.30) | 1.25 (.34) | .40 | .346 |
| Empathy (3-5 years) | .79 (58) | 1.07 (.26) | 1.07 (.28) | -.01 | .497 |
| Empathy (6-10 years) | .85 (60) | 1.35 (.27) | 1.42 (.29) | .98 | .165 |
| Negative emotion expression | .80 (123) | 2.42 (.55) | 2.43 (.54) | .16 | .437 |
| Positive emotion expression | .74 (123) | 3.65 (.66) | 3.63 (.54) | -.27 | .395 |
| Social competence | .68 (120) | 1.47 (.35) | 1.52 (.30) | .67 | .252 |
| Externalizing behaviors | .74 (121) | .72 (.39) | .62 (.30) | -1.50 | .066 |

*Note*: DHH = deaf and hard of hearing; TH = typically hearing.

^a^ Pooled results after multiple imputations.

^b^ One-tailed. Significance level was corrected for multiple testing to *p* < α/6 = .0083.

**Supplemental Table 5b**

*Hierarchical regression analyses for emotional functioning measures on social functioning after excluding the five children without a cochlear implant (pooled results after multiple imputations)*

|  | Social competence  (*N* = 124) | | | Externalizing behaviors  (*N* = 124) | | |
| --- | --- | --- | --- | --- | --- | --- |
|  | *b* | *p* | 95% *CI* | *b* | *p* | 95% *CI* |
| ***Step 1*** | *R*^2^ = .24** | | | *R*^2^ = .25** | | |
| Age | < .001 | .921 | [-.003, .002] | .001 | .282 | [-.001, .004] |
| Gender | -.001 | .990 | [-.11, .11] | .04 | .509 | [-.07, .15] |
| Group | -.04 | .481 | [-.14, .07] | .08 | .137 | [-.03, .20] |
| Emotion recognition | .06 | .210 | [-.03, .15] | -.01 | .859 | [-.10, .09] |
| Empathy | **.25** | **.022** | **[.04, .46]** | **-.32** | **.005** | **[-.55, -.10]** |
| Negative emotion expression | **-.18** | **< .001** | **[-.28, -.08]** | **.21** | **< .001** | **[.10, .31]** |
| Positive emotion expression | .01 | .872 | [-.09, .10] | .02 | .708 | [-.08, .12] |
|  |  |  |  |  |  |  |
| ***Step 2*** | ∆ *R*^2^ = .05 | | | ∆ *R*^2^ = .07* | | |
| Age |  |  |  | .002 | .130 | [-.001, .01] |
| Gender |  |  |  | .04 | .529 | [-.07, .14] |
| Group |  |  |  | -.61 | .188 | [-.53, .30] |
| Emotion recognition |  |  |  | .04 | .455 | [-.07, .15] |
| Empathy |  |  |  | **-.51** | **< .001** | **[-.77, -.25]** |
| Negative emotion expression |  |  |  | .05 | .441 | [-.08, .19] |
| Positive emotion expression |  |  |  | .04 | .540 | [-.09, .17] |
| Group x Emotion recognition |  |  |  | -.12 | .222 | [-.32, .07] |
| Group x Empathy |  |  |  | .38 | .072 | [-.03, .79] |
| Group x Negative emotion expression |  |  |  | **.33** | **.002** | **[.12, .54]** |
| Group x Positive emotion expression |  |  |  | -.05 | .638 | [-.24, .15] |

*Note*: Gender was coded as 0 = male, 1 = female. Group was coded as 0 = typically hearing, 1 = cochlear implant. 95% CI: 95% confidence interval. Independent variables were considered having an effect when *p* < α/2 = .025. **p* < .05; ***p* < .001 for the change in *R*^2^.

**Supplemental Table 6**

*Mean scores (standard deviations) per age group and comparisons between deaf and hard-of-hearing (DHH) children and children with typical hearing (TH)*

|  | Emotion recognition | Empathy | Negative emotion expression | Positive emotion expression | Social competence | Externalizing behaviors |
| --- | --- | --- | --- | --- | --- | --- |
| ***3-4 years*** |  |  |  |  |  |  |
| DHH (*n*=18) | 3.69 (.75) | 1.08 (.24) | 2.52 (.78) | 3.60 (.60) | 1.42 (.43) | .79 (.35) |
| TH (*n*=21) | 3.62 (.64) | 1.07 (.28) | 2.46 (.47) | 3.73 (.59) | 1.48 (.30) | .70 (.31) |
| *t* value^a^ | -.34 | -.11 | -.28 | .68 | .45 | -.82 |
| *p* value^ab^ | .366 | .457 | .390 | .250 | .327 | .208 |
| ***5-6 years*** |  |  |  |  |  |  |
| DHH (*n*=19) | 3.79 (.73) | 1.18 (.34) | 2.32 (.41) | 3.57 (.57) | 1.46 (.31) | .65 (.38) |
| TH (*n*=35) | 3.62 (.86) | 1.25 (.33) | 2.41 (.62) | 3.56 (.51) | 1.54 (.28) | .60 (.26) |
| *t* value^a^ | -.84 | .74 | .61 | -.24 | .94 | -.57 |
| *p* value^ab^ | .200 | .231 | .272 | .407 | .175 | .286 |
| ***7-10 years*** |  |  |  |  |  |  |
| DHH (*n*=18) | 3.64 (.48) | 1.37 (.21) | 2.31 (.43) | 3.77 (.74) | 1.54 (.34) | .71 (.42) |
| TH (*n*=18) | 3.59 (.63) | 1.45 (.30) | 2.43 (.44) | 3.64 (.56) | 1.51 (.32) | .57 (.34) |
| *t* value^a^ | -.25 | .89 | .82 | -.59 | -.22 | -1.03 |
| *p* value^ab^ | .402 | .187 | .208 | .278 | .415 | .152 |

^a^ Pooled results after multiple imputations.

^b^ One-tailed. Significance level was corrected for multiple testing to *p* < α/6 = .0083.
